# Unraveling neutrophil– *Yersinia* interactions during tissue infection

**DOI:** 10.12688/f1000research.18940.1

**Published:** 2019-07-11

**Authors:** Joan Mecsas

**Affiliations:** 1 Department of Molecular Biology and Microbiology, 136 Harrison Ave, Tufts University School of Medicine, Boston, MA, 02111, USA

**Keywords:** Yersinia pestis, Yersinia enterocolitica, Yersinia pseudotuberculosis, neutrophils, polymorphonuclear cells, YadA, Ail, Invasin, type 3 secretion system, Yops, SKAP2

## Abstract

The human and animal pathogens *Yersinia pestis*, which causes bubonic and pneumonic plague, and *Yersinia pseudotuberculosis* and *Yersinia enterocolitica*, which cause gastroenteritis, share a type 3 secretion system which injects effector proteins, Yops, into host cells. This system is critical for virulence of all three pathogens in tissue infection. Neutrophils are rapidly recruited to infected sites and all three pathogens frequently interact with and inject Yops into these cells during tissue infection. Host receptors, serum factors, and bacterial adhesins appear to collaborate to promote neutrophil– *Yersinia* interactions in tissues. The ability of neutrophils to control infection is mixed depending on the stage of infection and points to the efficiency of Yops and other bacterial factors to mitigate bactericidal effects of neutrophils. *Yersinia* in close proximity to neutrophils has higher levels of expression from *yop* promoters, and neutrophils in close proximity to *Yersinia* express higher levels of pro-survival genes than migrating neutrophils. In infected tissues, YopM increases neutrophil survival and YopH targets a SKAP2/SLP-76 signal transduction pathway. Yet the full impact of these and other Yops and other *Yersinia* factors on neutrophils in infected tissues has yet to be understood.

## Introduction

The study of the Gram-negative bacterial pathogens *Yersinia pestis*, *Yersinia pseudotuberculosis*, and *Yersinia enterocolitica* has been at the forefront of cellular and molecular pathogenesis for over four decades. *Y. pestis*, a recently emerged pathogen evolved from *Y. pseudotuberculosis*, is the causative agent of bubonic and pneumonic plagues and has produced several pandemics in the past 10,000 years ^1^. These infections are associated with high mortality rates of 30% and over 90%, respectively, if not treated rapidly with antibiotics. Transmission of *Y. pestis* to cause bubonic plague occurs via flea bite into the intradermal skin layer, whereas transmission of pneumonic plague occurs via inhalation of *Y. pestis* into lungs. By contrast to the highly lethal infections caused by *Y. pestis* in humans, *Y. pseudotuberculosis* and *Y. enterocolitica* generally cause self-limiting gastroenteritis and mesenteric lymph adenitis in most otherwise-healthy humans, rarely spreading to cause systemic disease or fatal infections. Infections normally occur through ingestion of contaminated foods or liquids. All three *Yersinia* species infect a variety of mammals, including rodents and ungulates, and the enteric pathogens can be found in birds. Thus, the *Yersiniae* are “generalists”, adept at surviving in many different hosts, and so have evolved virulence factors and pathogenic strategies that counteract immune systems of a variety of animals.

Over the past 35 years, the study of the *Yersinia* virulence factors—including bacterial adhesins, the type 3 secretion system (T3SS), effector proteins (Yops), Pla protease in *Y. pestis*, and iron acquisition systems—has revealed critical features of host–pathogen interactions (reviewed in 2– 5). Notable recent advances include uncovering aspects of innate immunity that are triggered or suppressed (or both) by the T3SS and Yops in macrophages ^6– 17^ and reconstructing the recent evolutionary progression from *Y. pseudotuberculosis* to *Y. pestis* ^1, 18– 20^. Another critical feature of *Yersinia*–host cell interaction garnering attention is its interactions with neutrophils during various types of tissue infection. Neutrophils are critical cells of the innate immunity system and both sense pathogens resulting in release of signaling molecules, such as cytokines and alarmins, and kill invading microbes through a variety of mechanisms ^21^. These killing mechanisms include phagocytosis, generation of reactive oxygen species, degranulation, and formation of neutrophil extracellular traps ^21– 23^. Effector Yops hamper a number of these processes in isolated neutrophils ^2, 3, 24– 30^, observations which are further driving current interest in how neutrophils interact with *Yersinia* in the context of infected tissues and other cell types. This mini-review highlights recent studies involving *Yersinia*–neutrophil interactions in murine tissues.

## *Yersinia* spps target Yop injection to neutrophils during infection of tissues

*Yersinia* spps use the highly conserved T3SS to inject six or seven effector Yop proteins in host cells to cause disease in mammals ^2, 5, 31^. In tissue infections using a β-lactamase reporter system, studies with *Y. pestis*, *Y. enterocolitica*, and *Y. pseudotuberculosis* have demonstrated that neutrophils are a major—and, typically, the primary—cell target for Yop injection ^32– 38^. That neutrophils are a significant target holds true regardless of whether the route of infection is oral, intravenous, or intranasal and the tissues examined are Peyer’s patches, mesenteric lymph nodes, spleens, or lungs ^32– 37^.

There are, however, several exceptions, most notably early after tissue infection ^32, 39, 40^. For example, 6 hours after intranasal infection with the virulent *Y. pestis* CO92 strain, alveolar macrophages are the primary injected cell type and comprise over 50% of injected cells whereas neutrophils comprise about 15% of the injected population ^36^. This balance shifts by 12 hours when neutrophils start invading tissues at higher numbers and become over 70% of the injected population ^36^. Similarly, 1 day after intravenous infection with *Y. enterocolitica*, macrophages are more highly targeted in the spleen but by day 2 neutrophils become equally targeted ^39^. After oral infection with *Y. pseudotuberculosis*, levels of injected macrophages and neutrophils in the mesenteric lymph nodes are comparable 5 days after oral gavage ^32^. Finally, in splenic infections, while neutrophils are enriched significantly for injection, frequently a comparable or even higher absolute number of B cells are injected with Yops ^32, 33, 35, 39^. The high injection levels of B cells may occur because they comprise the majority of total cells in spleens—over 60% compared with the much lower numbers of neutrophils and macrophages—so *Yersinia* may stochastically encounter them more than any other cell type ^32, 33, 35^. For insights into the Yop interactions with B and T cells, readers are referred to the following ^38, 41– 44^.

## What factors are important for *Yersinia* targeting Yops into neutrophils?

### Proximity

There are both physiological and molecular explanations for why neutrophils are a major target of *Yersinia* Yop injection during infection. One significant reason is location of the cells to the bacteria since tight binding of *Yersinia* to cells is required for Yop injection ^25, 45, 46^, and neutrophils migrate to inoculation sites within hours or days after infection and are typically the closest cell type associated with the bacteria. After infection with the enteric *Yersinia* pathogens, neutrophils migrate to and eventually encase the bacterial colonies in the Peyer’s patches or spleens between 24 and 72 hours after oral or intravenous infection ^47– 49^. In these tissues, pyogranulomas form containing tightly packed *Y. pseudotuberculosis* immediately surrounded by neutrophils with macrophages forming an outer ring of cells ^47^. It is noteworthy that, in some cases, neutrophils associate more rapidly with *yop* mutants than with wild-type *Yersinia*. For instance, within a day after oral inoculation with wild-type *Y. pseudotuberculosis*, *Y. enterocolitica*, or *yop* mutants, more neutrophils are found in association with the *yop* mutants than the wild-type *Yersinia*, indicating that early interactions of wild-type *Yersinia* with resident tissue cells delay chemotaxis of neutrophils to the bacteria ^48, 49^.

After intradermal inoculation, by either a flea bite or needle inoculation, neutrophils are detected within 50 minutes to 7 hours of inoculation ^50– 52^. Likewise, in lung infections with *Y. pestis* or *Y. pseudotuberculosis*, neutrophils migrate to tissue sites within 12 to 48 hours, depending on the strain ^36, 37, 53, 54^.

Yet several lines of evidence suggest that proximity is not the only factor critical for the preponderance of neutrophils found injected with Yops. In one study, in which neutrophils and inflammatory monocytes were depleted from tissues, fewer overall cells are targeted for injection rather than different cell populations ^32^. This suggests that the nature of bacterial growth within tissues or interactions with the remaining innate immune cells in tissues (or both) play a role in the number of injected cells during tissue infection.

### Receptors, adhesins, and serum factors

*Ex vivo* studies show that the selectivity for injection into neutrophils is recapitulated in single-cell splenic and lung homogenates infected with *Yersinia* when bacteria are limiting, indicating that there are specific receptor–ligand interactions that are favored between the *Yersinia* and neutrophils ^32, 34, 35, 37, 39, 55^. Blockage of complement receptor 3 (CR3) on neutrophils from *ex vivo* splenic homogenates significantly reduced injection into neutrophils by *Y. pestis* in splenocytes, demonstrating that *Y. pestis* uses this receptor ^55^ and suggesting that the CR3 receptor, which is enriched in neutrophils, plays a role in promoting *Y. pestis*–neutrophil interactions over other cell types in these lysates. This has yet to be evaluated in a mouse model of infection with *Y. pestis*. But findings with *Y. pseudotuberculosis* also show a role for complement or serum factors in directing injection of Yops into neutrophils in isolated mouse splenocytes and in mouse infections in spleens after intravenous but not in intranasal infections ^34, 37^. Elegant *in vivo* studies with β1-depleted mice demonstrate that *Y. enterocolitica* uses β1 integrins to inject Yops into many different cell types in infected spleens, although this receptor usage was not specific to neutrophils ^39^.

The enteric *Yersinia* bacterial adhesins YadA, Invasin, or Ail (or a combination of these) are important for injection in mouse tissues ^34, 39^. Under conditions where adhesin-mutants and the wild-type strain were recovered at comparable numbers, a Δ *ail*Δ *inv*Δ *yadA* triple mutant in *Y. pseudotuberculosis* and a *yadA* mutant in *Y. enterocolitica* injected fewer cells after intravenous infection than the isogenic wild-type strains ^34, 39^. In the case of *Y. enterocolitica*, very few neutrophils are detected in tissues correlating with very few injected cells and this is similar to findings with *Y. pseudotuberculosis* ^32, 39^. After infection with the Δ *ail*Δ *inv*Δ *yadA* triple mutant in *Y. pseudotuberculosis*, the spectrum of injected cells was not changed ^34^. However, treatment with cobra venom factor both restores virulence of the triple mutant and causes a significant shift in spectrum of cells targeted by the triple-mutant but not the wild-type strain ^34^. Specifically, in mice treated with cobra venom factor (which is a complement-activating protein that depletes complement regulatory proteins and ultimately complement), the triple mutant injects more B cells and fewer neutrophils ^34^. This result shows that a combination of serum factors and bacteria adhesins influences cells targeted for injection in *Y. pseudotuberculosis*. However, it remains to be determined whether the nature of the pyogranuloma formed under these conditions is the same as or different from that formed by the wild-type strain ^47^ and whether this explains the altered spectrum of injected cells. Nonetheless, Ail and YadA have long been recognized in *in vitro* studies to interact with serum factors and to bind cells and promote injection ^45, 56– 62^; these *in vivo* studies demonstrate their importance for injection of Yops in tissue infection.

## Do neutrophils matter in tissue infection?

Given that *Yersinia* expends energy injecting Yops into neutrophils and appears to have co-opted CR3 or serum factors (or both) in mouse tissues to enhance injection into neutrophils, the question arises “Are neutrophils important to contain *Yersinia* infection?” If neutrophils are important, one would predict increased colony-forming units (CFUs), increased disease symptoms, and decreased time to morbidity in their absence. At first glance, the results of the classic approach of depleting neutrophils and measuring infection outcomes are mixed.

In intradermal models of infected mice depleted of neutrophils with 1A8, an antibody recognizing Ly6G found on mature neutrophils ^63^, the CFUs of *Y. pestis* in the skin increase significantly in the absence of neutrophils, yet the total number of bacteria replicating in the draining lymph node does not change ^64^. Likewise, CFUs of *Y. pestis* in lymph tissues remain constant after depletion with RB6-8C5 ^52^, an antibody that depletes Gr-1–expressing cells, including mature and immature neutrophils and subsets of inflammatory monocytes, dendritic cells, and T cells ^65, 66^. These results support the ideas that neutrophils are important for curbing bacterial growth in the skin, but not the lymph nodes, and that neutrophils are not essential for dissemination from skin to lymph tissues. Some debate exists about whether *Y. pestis* disseminates from skin to lymph nodes by hitchhiking in neutrophils, macrophages, or dendritic cells or a combination of these (reviewed in 67). But these most recent studies support the idea that *Y. pestis* can disseminate to lymph nodes independently of neutrophils.

Depletion of neutrophils with 1A8 in lung infection with *Y. pestis*, as with lymph node infection, does not result in changes in bacteria counts 24 or 48 hours after infection ^36^. Surprisingly, symptoms of disease progression and time to death are reduced in the absence of neutrophils, although the difference in time to death is not statistically significant ^36^. Consistent with these findings, the histopathology of mice treated with 1A8 showed less damage to lungs with intact alveoli structure whereas untreated mice had necrotizing pneumonia ^36^. However, artificially increasing the numbers of neutrophils in lungs prior to infection significantly attenuates *Y. pestis* infection ^53^. Combined, these results show that, early after infection, high levels of neutrophils stop infection, but once *Y. pestis* reaches a certain stage—either in number or in modulating early host responses or both—the bactericidal activities of neutrophils are effectively nullified and, in fact, their continued migration into lungs wrought the damage observed during infection.

The impact of neutrophils on restraining infection by the enteric *Yersinia* pathogens is equally mixed. In an oral infection model of *Y. pseudotuberculosis*, depletion of neutrophils with 1A8 or RB6-8C5 resulted in significantly more growth at day 1 post-infection with wild-type and *yopE*, *yopH*, and *yopK* mutant strains, and disease symptoms were worse on subsequent days ^48, 68^. Likewise, increasing the numbers of neutrophils in Peyer’s patches or increasing their activation in spleens by depletion of dendritic cells results in increased clearance of wild-type *Y. enterocolitica* ^49, 69^. Increasing neutrophils in tissues also suppresses *Y. pseudotuberculosis yop* mutant colonization but not wild-type colonization in oral infection ^70, 71^. Although these results point to some differences between the enteric *Yersiniae* interactions with neutrophils, they indicate that neutrophils can control early seeding or dissemination events in infection (or both). However, neutrophil depletion does not always increase *Y. pseudotuberculosis* growth in tissues. Three days after oral inoculation, fewer wild-type *Y. pseudotuberculosis* were detected overall in neutrophil-depleted mice (by luminescence) compared with non-depleted tissues despite worsening disease symptoms ^48, 68^. After intravenous infection with *Y. pseudotuberculosis*, the number of bacteria recovered 3 days from mock-depleted, 1A8-treated or RB6-8C5–treated mice was comparable despite the observation that 1A8- or RB6-8C5-treated mice appeared more ill and reached morbidity faster ^26^. Thus, although the growth of *Y. pseudotuberculosis* is not always increased in the tissues examined in the absence of neutrophils, the overall health of the mice typically worsens.

Overall, these results are consistent with the idea that *Yersinia* handles intimate interactions with neutrophils effectively once infection in a tissue is established, but the bacteria are more susceptible to neutrophils early in infection, such as soon after inoculation or when disseminating to new tissues. Supporting the idea that *Yersinia* spps are well designed to withstand neutrophil onslaught in tissues is the observation that a number of attenuated *Yersinia* mutants grow significantly better in neutrophil-depleted mice, indicating that the function of these proteins is to inactivate neutrophils or withstand the bactericidal activities of neutrophils. Importantly, adhesin mutants *yadA* and *ail* and several *yop* mutants such as *yopH*, *yopE*, and *yopK* mutants ^26, 37, 48, 72^ all colonize significantly better in some tissues in neutrophil-depleted mice than in wild-type mice. (Not every mutant is restored for growth in the absence of neutrophils ^73, 74^; for example, some are restored in the absence of both neutrophils and inflammatory monocytes ^73, 75^ and some have been tested only in the absence of both ^75^.)

## What are the consequences to neutrophils after Yop injection in mouse infections?

Several elegant studies have examined the transcriptome of cells surrounding *Yersinia* microcolonies by using RNA sequencing (RNA-seq) ^76, 77^. When dual-tissue RNA-seq was used to evaluate the host cell and bacterial responses to infection of *Y. pseudotuberculosis* in the Peyer’s patches, a number of host transcripts associated with infection were strongly induced; this is indicative of the pronounced neutrophil infiltrate that occurs after infection ^43, 48, 77^. These included metal ion sequestration, inflammatory responses, acute-phase responses, and coagulative activities ^77^. These findings shed light into the overall host response to infection in tissues which are composed predominately of neutrophils, but the findings do not distinguish the cells specifically in contact with bacterial microcolonies. The β-lactamase reporter system ^32, 33, 35^ can also be used to distinguish and isolate injected from non-injected neutrophils in infected tissues. Via such an approach, YopH, a tyrosine phosphatase, was found to target the Slp-76/SKAP-2/PRAM pathway in neutrophils during tissue infection ^26^. This pathway is critical for reactive oxygen production of neutrophils after integrin stimulation ^78^, providing a possible role for YopH. This approach can be further exploited to determine direct from indirect consequences of *Yersinia*–neutrophil interactions in tissues.

Via laser capture microdissection, the inflammatory lesions in the lungs induced by *Y. pestis* were parsed on the basis of proximal (and presumably containing many cells injected with Yops) and distal areas to *Y. pestis* microcolonies ^76^. These transcriptomes were compared with each other and with the transcriptome of bone marrow neutrophils from uninfected (representing not activated) and infected mice. Remarkably, the transcriptomes of cells proximal to the bacteria are most similar to bone marrow neutrophils from uninfected mice; that is, both resemble non-activated cells with higher expression of pro-survival signals and lower expression of chemotaxis/migration genes than the more distally located cells ^76^. Strikingly, YopM expression changes the physiology of neutrophils in tissue infection but not the bacterial burden. In histological sections of mice infected with a *yopM* mutant, cells appear anucleated and express more apoptotic markers ^76^, indicating that YopM contributes to the pro-survival state of the neutrophils yet this is not sufficient to impact bacterial survival.

## What are the consequences to *Yersinia* after contact with neutrophils in mouse tissues?

Changes to *Y. pseudotuberculosis* and *Y. pestis* gene expression in lymph tissues that contain high numbers of neutrophils have been analyzed by microarray analysis and RNA-seq, respectively ^77, 79^. Notably, genes required for metal ion acquisition, nitric oxide (NO) stress responsiveness, and (in *Y. pseudotuberculosis*) carbohydrate use were mostly highly upregulated in tissues compared with 37°C broth-grown cultures ^77, 79^. Many of these pathways are also critical for survival of *Y. pestis* in a rat buboe model ^80^. Collectively, these results point toward a local lymph environment where the bacteria experience high NO stress and restrictive ion and metabolic conditions. *Y. pestis* and *Y. pseudotuberculosis* respond to this restrictive metabolic environment in different ways; *Y. pseudotuberculosis* induces carbohydrate use genes and the upper part of glycolysis, whereas *Y. pestis* uses anaerobic respiration ^77, 79, 80^.

Direct observation of *Y. pseudotuberculosis* expressing fluorescent reporter constructs that are responsive to different environmental cues has permitted further dissection of bacterial responses in tissues ^47^. Specifically, at the periphery of microcolonies, *Y. pseudotuberculosis* expresses higher levels from the *hmp* promoter, an NO responsive gene, and *yopE*, a T3SS gene. Higher NO expression is more uniformly observed in the outer ring of the microcolony, yet inducible nitric oxide synthase (iNOS) was not detected in the immediately adjacent cells, indicating that NO diffuses from a distance ^47^. By contrast, high expression from the *yopE* promoter was sporadically observed in individual cells on the periphery, suggesting that these cells are in direct contact with neutrophils and therefore upregulating *yopE* transcription ^47^. These findings are consistent with increased copy number of the plasmid containing the T3SS upon contact with host cells ^81^.

## Emerging models: *Yersinia*–neutrophil interactions in murine tissues

Many facets of *Yersinia*–neutrophil interactions have yet to be unraveled, but a working model of *Yersinia*–neutrophil interactions in infected tissues is beginning to emerge. Neutrophils are recruited rapidly to infected tissues, albeit sometimes after a delay relative to recruitment by a *yop* mutant. This delay suggests that very early Yop injection into resident tissue cells may modulate chemokine and cytokine release, delaying neutrophil recruitment. However, neutrophils rapidly become the most proximal cell type to *Yersinia*, surround them, and in turn are efficiently injected with Yops by *Yersinia*. Higher expression of stress response genes and T3SS promoters is observed in line with increases in copy number of the pYV plasmid and increases in ion sequestration genes in host cells. Injection disarms neutrophils without triggering significant cell death. Rather, the immediately adjacent cells adopt a pro-survival and low migration state that fails to reduce bacterial growth.

Understanding how different Yops collaborate to modulate neutrophil activities in tissue infection is ongoing. Because it is easier to obtain human primary neutrophils in large quantities relative to mice, most studies investigating *Yersinia*–neutrophil interactions have used isolated human neutrophils ^24, 29, 82– 86^. It is important to be aware that murine and human neutrophils have notable differences and thus findings in one system cannot be inferred to occur in another ^87, 88^. Furthermore, human neutrophils are typically harvested from peripheral blood whereas mouse neutrophils are collected from either the bone marrow or the peritoneal cavity after being elicited by an irritant, such as casein or thioglycolate. Therefore, these cells are in different stages of development and have the potential to respond to bacteria differently. Nonetheless, studies in either system are important to understand how *Yersinia*, through manipulation of neutrophils, thwarts the orchestrated mammalian host cell response to infection at an organismal, tissue, cellular, and molecular level.
